# Classifying voice disorders for machine learning: a pilot study using the USVAC-C2025 diagnostic framework

**DOI:** 10.3389/fdgth.2026.1752356

**Published:** 2026-06-23

**Authors:** Catherine Madill, Zhou Hao Leong, Dharshini Manoharan, Dhanshree Gunjawate, Charu Grover, Katrina Sandham, Rijul Gupta, Craig Jin, Duy Duong Nguyen, James Jordan Johnson, Daniel Novakovic

**Affiliations:** 1 Voice Research Laboratory, Faculty of Medicine and Health, University of Sydney, Sydney, NSW, Australia; 2 Faculty of Medicine, Dai Nam University, Hanoi, Vietnam

**Keywords:** diagnostic classification, inter-rater reliability, machine learning, videostroboscopy, voice disorders, artificial intelligence

## Abstract

**Introduction:**

Machine learning for voice disorders relies heavily on accurate diagnostic classification, yet progress has been limited by inconsistent labelling and the absence of a reproducible framework suitable for clinical and computational use. This study aimed to develop and evaluate a multilayer classification system for voice disorder diagnosis tailored for machine learning applications, and to determine its inter- and intra-rater reliability among otolaryngologists and speech-language pathologists.

**Method:**

We conducted a diagnostic reliability study of 45 adults with voice disorders who underwent comprehensive clinical assessment, including videostroboscopy, at a tertiary voice clinic in Sydney, Australia, between February 2018 and March 2024. A multidisciplinary team developed a five-level hierarchical classification framework through iterative consensus. Four blinded raters independently applied the framework to anonymised video and clinical datasets, with 15 cases randomly repeated for intra-rater analysis. Reliability was quantified using Fleiss *κ* statistics and intraclass correlation coefficients across all diagnostic levels.

**Results:**

Intra-rater reliability was high (intraclass correlation coefficient range, 0.768–0.865), with comparable consistency across disciplines. Inter-rater reliability was strongest for identifying disordered vs. non-disordered voices (*κ* = 0.812; 95% CI, 0.733–0.891) and major aetiological categories (*κ* = 0.695; 95% CI, 0.611–0.779), supporting the utility of structured classification for foundational diagnostic decisions. Agreement declined with increasing diagnostic specificity, particularly for perceptually based conditions such as muscle tension disorders (*κ* = 0.253; 95% CI, 0.172–0.334) and vocal fold paresis (*κ* = 0.238; 95% CI, 0.155–0.321). Functional neurological voice disorders and structural lesions demonstrated the highest category-level agreement.

**Conclusion:**

These findings show that a structured, multilayer framework improves diagnostic consistency where machine learning systems most rely on stable labels and highlights key areas of diagnostic ambiguity. The system provides a practical foundation for creating reliable annotated datasets and supports future development of machine learning tools for voice disorder classification and clinical decision support.

## Introduction

### Barriers to the translation of automatic voice disorder detection

Machine learning (ML) has generated increasing interest in voice diagnostics with growing evidence that algorithmic models can accurately distinguish between normal and disordered voices using acoustic, aerodynamic, and visual data (1–4). These tools can improve access to expert-level diagnosis, enhance clinical efficiency, and support triage in under-resourced settings. However, despite strong reported performance in research contexts, ML systems for voice disorders have seen minimal clinical integration (2, 5–7). This translational gap appears to stem not only from variability and inconsistency in the diagnostic inputs provided by clinicians but also from the limited availability of labelled data and the heterogeneity of diagnostic categories used across institutions (1, 2, 8, 9).

Supervised ML models rely on reproducible diagnostic input. Yet many voice disorders, especially those that are perceptually defined, multifactorial, or lack clear objective markers, are diagnosed inconsistently across clinicians and institutions (9). This heterogeneity in how voice assessments are conducted, interpreted, and summarised introduces inconsistency into training datasets and limits the ability of ML models to generalise reliably. Addressing this issue requires more than refining algorithm design; it necessitates standardising the diagnostic frameworks and processes that underpin the assessment of voice disorders (2).

### There is no universally accepted system for classifying voice disorders

A core obstacle to diagnostic standardization is the lack of a universally accepted classification framework that is clinically valid and compatible with ML pipelines. Several systems have been proposed, including the Classification Manual for Voice Disorders–I (CMVD-I) (10), the Morrison and Rammage framework (11), Baker's modified diagnostic classification system (12, 13), and, more recently, the hierarchical system proposed by Payten et al. (9). Each offers distinct perspectives but differs in structure, terminology, and diagnostic granularity. This inconsistency makes it difficult to harmonize diagnostic practices across clinicians, institutions, or studies, hindering the development of generalizable ML models (2, 4).

### Reasons for the voice disorder classification challenge

The challenge of classification is compounded by the multifactorial nature of voice disorders and the limitations of existing diagnostic tools. Many voice disorders present with overlapping symptoms, fluctuating severity, and no definitive biomarker (14, 15). Conditions such as muscle tension voice disorder (MTVD), vocal fold paresis, and functional neurological dysphonia often rely on clinician judgment, perceptual voice assessment, and inference from stroboscopic findings rather than clearly defined objective criteria. Even among experts, such diagnoses are applied inconsistently. Prior studies have shown that inter-rater reliability for perceptual voice assessments can be moderate at best, particularly for conditions without overt structural pathology (16–18). These inconsistencies introduce unwanted variability into clinical care and research datasets, presenting a significant obstacle for supervised ML training.

### Developing a robust multilayer diagnostic framework for ML

To address these challenges, we developed a multilayer diagnostic classification framework that is structured, clinically grounded, and optimised for ML research. The system was designed through an iterative consensus process involving laryngologists, speech-language pathologists, and ML experts. This process organises voice disorders hierarchically across five levels of diagnostic specificity (0–4), from binary classification (normal vs. abnormal) to specific clinical entities (e.g., Reinke's oedema, vocal fold paresis). This structure allows clinicians to assign labels based on diagnostic certainty and available information, while also providing a scaffold for ML models to perform hierarchical classification tasks.

The multilayer design also facilitates interoperability and consistency across users. It provides a shared taxonomy that can be applied in clinical and research settings, enabling systematic annotation of voice disorder datasets. Critically, it reflects how clinicians think about voice disorders. First, it establishes that a disorder is present, then infers its broad etiology, and finally, narrows it down to a specific diagnosis. The framework enhances clinical transparency and supports computational interpretability by mirroring this reasoning process.

### Diagnosis needs to be based on a multidimensional assessment

Recognising the complexity of voice disorder diagnosis, the framework incorporates a multidimensional assessment approach that includes patient history, patient-reported outcome measures (PROMS), auditory-perceptual evaluation, acoustic and aerodynamic analysis, and videostroboscopic findings. This reflects best practices in contemporary voice clinics, where diagnosis is typically based on integrating evidence across domains. The system allows for diagnostic labeling even in cases where only partial information is available, which is critical for both retrospective data annotation and prospective ML deployment.

**Figure 1:**
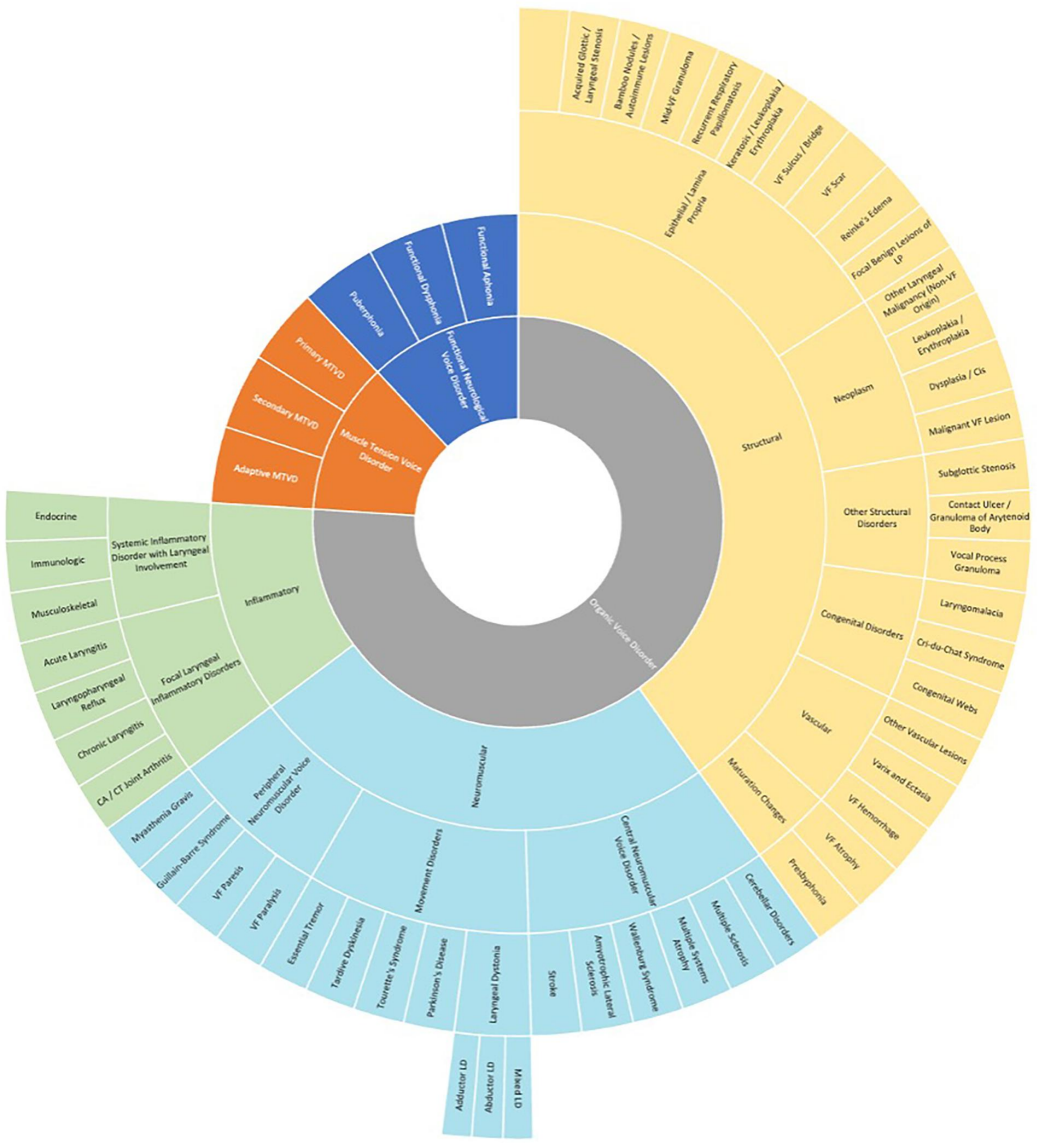
A five-level hierarchical classification schema for voice disorders. Level 0 distinguishes normal from abnormal voice. Level 1 assigns etiological class (organic, functional neurological, or muscle tension). Level 2 subdivides organic cases into structural or neuromuscular categories. Levels 3 and 4 define diagnostic groupings and specific diagnoses, respectively. Examples shown at Levels 3 and 4 are not exhaustive and represent a subset of possible diagnostic categories. This structure supports standardised data labelling for clinical and machine learning use.

The framework's five diagnostic levels are: (0) normal vs. abnormal; (1) broad etiological category (organic, functional neurological, or muscle tension); (2) organic subtype (structural or neuromuscular); (3) diagnostic grouping (e.g., benign lesions, inflammatory disorders); and (4) specific diagnosis (e.g., nodules, vocal fold paralysis, MTD) (Figure 1). This structure accommodates the spectrum of diagnostic confidence and complexity encountered in real-world clinical practice.

### Study objective

The primary aim of this study was to evaluate the inter- and intra-rater reliability of this diagnostic classification framework when applied to real-world patient data by trained otolaryngologists and speech-language pathologists. We hypothesised that reliability would be highest at the upper levels of the hierarchy (e.g., normal vs. disordered; organic vs. functional) and lower at more granular levels (e.g., specific diagnoses), particularly for perceptually defined categories such as MTVD and vocal fold paresis. By establishing the reproducibility of this system, we aim to lay the groundwork for consistent data annotation in ML research and to advance clinical standardisation in the diagnosis of voice disorders.

## Methods

The study was approved by the University of Sydney Human Research Ethics Committee (Project No. **2022/825**). This study followed the STROBE reporting guideline.

### Classification framework development

A multidisciplinary team of three Otolaryngologists (ORL), four speech-language pathologists (SLP), and two ML specialists developed the classification framework through an iterative, three-stage consensus process. Existing classification models, including the Classification Manual for Voice Disorders–I, Morrison and Rammage's system, Baker's framework, and Payten's hierarchical model, were reviewed to inform the structure and terminology of the new framework. The five nested diagnostic layers addressed key limitations in diagnostic clarity, interoperability, and ML suitability (Supplementary Table S1). The system was piloted on test cases and refined until all raters agreed on definitions and applications.

### Reliability assessment of the classification framework

#### Data for reliability ratings

We retrospectively identified 50 patients from the University of Sydney Voice Assessment Clinic, evaluated between February 2018 and March 2024. All patients had consented to use their deidentified clinical data for research purposes. Patients were drawn from an institutional clinical database of 155 individuals seen in the voice clinic during this period by an independent investigator. The included patients had complete stroboscopic recordings and comprehensive clinical assessments. Patients with incomplete datasets were excluded. Forty-five patient cases were selected to ensure representation across all major diagnostic categories in the framework, including organic-structural, organic-neuromuscular, functional neurological, and primary muscle tension voice disorders. All included patients were confirmed to have a voice disorder diagnosis. The demographic and diagnostic characteristics of the 45 patients included are summarized in Table 1.

**Table 1:** Demographic and clinical characteristics of patient cohort.

Characteristic	Value
Number of patients	45
Mean age (years)	42.7
Age range (years)	18–78
Sex: Female	26 (57.8%)
Sex: Male	19 (42.2%)
Functional Neurological Voice Disorder	6 (13.3%)
Muscle Tension Voice Disorder	11 (24.4%)
Organic–Structural Disorder	19 (42.2%)
Organic–Neuromuscular Disorder	9 (20.0%)

Summary of age, sex distribution, and diagnostic categories among the 45 patients evaluated using the classification system.

All included patients had undergone a standardized, multidisciplinary voice assessment protocol. Their assessment protocols included case history questionnaire (demographic information, symptom history, medical comorbidities, and medication profiles); patient-reported outcome measures (Voice Handicap Index-10, Cough Severity Index, Reflux Symptom Index, and Glottal Function Index); SLP's auditory-perceptual assessment (CAPE-V) (19); acoustic voice analysis; aerodynamic assessment using Pentax Medical's Phonatory Aerodynamic System (PAS); and flexible laryngeal videostroboscopy. The stroboscopy videos were collected during examination by fellowship-trained laryngologists using a high-definition digital system (Pentax EPK-3000). During stroboscopy examination, the patients completed a fixed sequence of tasks, including sustained vowels at low, modal, and falsetto pitch, pitch glides, staccato syllables, the sniff maneuver, and connected speech samples (20). Recordings were archived with synchronous audio and served as the visual-perceptual component of each case. The complete dataset for diagnosis for each patient included past medical history, current voice symptoms, laryngeal imaging, auditory-perceptual features, and acoustic measures.

#### Rater training and blinding

Four clinicians from the same research group (authors ZH, DN, CM, and DM) served as independent raters for the initial evaluation of the classification system: two otolaryngologists specializing in laryngology and two SLPs with expertise in voice disorders. All raters attended a structured training session on the classification framework, during which they reviewed definitions, example cases, and decision-making rules. A research assistant anonymized all patient datasets and assigned random identifiers. Raters were provided only with age, sex, and relevant medical history for each case (sourced from the assessment report). They were blinded to the patient's original diagnoses and each other's ratings.

#### Videostroboscopic image rating procedures

Materials for ratings included assessment data (stroboscopy video with sound, and past medical history) of 45 patients and 15 duplicates for reliability analysis. All samples (*n* = 60) were uploaded into an online rating tool called Bridge2Practice.com, where the classification framework rating form had been created (21). The link to the online tool was then sent to each rater, who was required to classify the samples in random order using the framework mentioned above. Duplicate files were indistinguishable from originals and were presented in a randomized order. Rating data were exported as an Excel spreadsheet for data analysis.

### Statistical analysis

All statistical analyses were conducted using SPSS version 28.0 (IBM Corp., Armonk, NY). The intra-rater reliability of each diagnostic level was calculated using Fleiss' kappa for categorical agreement and intraclass correlation coefficients (ICC) for consistency. ICC values were computed using a two-way random-effects model, single measurement [ICC (1, 2)] for the repeated ratings. Reliability was interpreted using established thresholds: values >0.80 indicated good agreement, 0.61–0.8 good, 0.41–0.6 moderate, and 0.21–0.4 fair agreement. In all calculations, a significance level of *p* < 0.05 was used.

## Results

All 60 cases (including duplicate cases) were classified across five diagnostic layers, resulting in 300 individual case-level assessments per rater. All four raters completed the full rating protocol without missing data. Duplicate cases showed consistent label assignment across rating sessions in most instances.

### Intra-rater reliability

The ICC values from the 15 repeated cases ranged from 0.768 to 0.865 across the four raters, indicating good test-retest consistency. The two laryngologists showed slightly higher ICCs (mean 0.842) than the two speech-language pathologists (mean 0.781), though this difference was not statistically significant. Cases with the lowest intra-rater reliability across all four raters reported subtle signs of vocal fold motion asymmetry or hyperfunction without overt structural abnormalities in the assessment report findings. Pairwise agreement across raters was highest between Rater 1 and Rater 3 (71%), with the lowest agreement observed between Rater 2 and Rater 3 (65%).

### Inter-rater reliability

**Figure 2:**
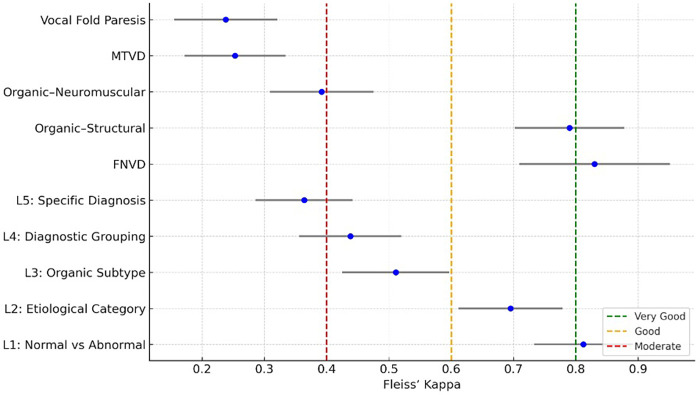
Inter-rater reliability by diagnostic layer and category. Fleiss' kappa values (with 95% confidence intervals) for inter-rater agreement across each hierarchical diagnostic level and selected voice disorder categories. Agreement was highest for functional neurological voice disorders and organic–structural diagnoses. Substantial variability was observed in categories relying on perceptual judgment, such as muscle tension voice disorder, and vocal fold paresis. Vertical dashed lines denote threshold values for interpretive reliability: >0.80 (very good), 0.61–0.80 (good), and 0.41–0.60 (moderate).

Inter-rater reliability was uniformly lower than intra-rater reliability. Inter-rater reliability varied across diagnostic layers and was highest at the broadest classification level (non-disordered vs. disordered). Fleiss' kappa was 0.812 (95% CI: 0.733–0.891) for the binary classification of non-disordered vs. disordered voice, indicating very good agreement. Inter-rater reliability remained good at the primary etiological level (organic, functional neurological, or muscle tension), with *κ* = 0.695 (95% CI: 0.611–0.779). However, the reliability decreased with increasing diagnostic specificity. At the third level, i.e., differentiating between organic-structural and organic-neuromuscular subtypes, *κ* dropped to 0.511 (95% CI: 0.425–0.597), reflecting moderate agreement. At the diagnostic grouping level, *κ* = 0.438 (95% CI: 0.356–0.520), and at the final specific diagnosis level, agreement was fair, with *κ* = 0.364 (95% CI: 0.286–0.442). Fleiss' kappa values by diagnostic level and diagnostic category are shown in Table 2. These patterns are further illustrated in Figure 2, which shows inter-rater agreement declining with increasing diagnostic granularity.

**Table 2:** Inter-rater reliability results by diagnostic level and category.

Diagnostic Level/Category	Fleiss' Kappa (95% CI)
L0: Normal vs. Abnormal	0.812 (0.733–0.891)
L1: Etiological Category	0.695 (0.611–0.779)
L2: Organic Subtype	0.511 (0.425–0.597)
L3: Diagnostic Grouping	0.438 (0.356–0.520)
L4: Specific Diagnosis	0.364 (0.286–0.442)
Functional Neurological Voice Disorder	0.830 (0.709–0.951)
Organic–Structural Disorder	0.790 (0.702–0.878)
Organic–Neuromuscular Disorder	0.392 (0.309–0.475)
Muscle Tension Voice Disorder	0.253 (0.172–0.334)
Vocal Fold Paresis	0.238 (0.155–0.321)

Fleiss' kappa values with 95% confidence intervals and intra-rater intraclass correlation coefficient (ICC) ranges for each classification level and major diagnostic category.

Functional neurological voice disorders demonstrated the highest diagnostic agreement across all levels, with *κ* = 0.830 (95% CI: 0.709–0.951). Structural organic disorders also yielded strong inter-rater reliability (*κ* = 0.790, 95% CI: 0.702–0.878), particularly for lesions such as cysts, nodules, and polyps, where visual stroboscopic findings were easily identifiable. In contrast, muscle tension voice disorders demonstrated poor agreement (*κ* = 0.253, 95% CI: 0.172–0.334), and organic-neuromuscular disorders, including vocal fold paresis, showed similarly low reliability (*κ* = 0.238, 95% CI: 0.155–0.321). When comparing rates by discipline, no significant differences in inter-rater reliability were observed between otolaryngologists and speech-language pathologists. Across all diagnostic layers, the mean *κ* for otolaryngologist dyads was 0.593, and for speech-language pathology dyads was 0.579. Cross-disciplinary dyads showed similar agreement (mean *κ* = 0.582), suggesting that the classification system was applied similarly across clinical specialties.

### Diagnostic frequency

Analysis of etiological diagnostic classification (L2) frequency revealed that organic-structural disorders were the most assigned category, comprising 42.7% of total ratings. Muscle tension voice disorders accounted for 24.3%, organic-neuromuscular disorders for 18.9%, and functional neurological disorders for 14.1%. The distribution of lower-level classifications was broadly consistent across raters. However, some variability in subcategory assignment was noted, particularly in cases with overlapping features such as co-occurring tension and paresis. Assignment frequencies across etiological diagnostic categories are presented in Table 3, with organic–structural disorders most commonly labeled.

**Table 3:** Frequency of diagnoses assigned across all raters.

Etiological Category (L2)	Number of Assignments	Percentage of Total (%)
Organic–Structural	77	42.8
Organic–Neuromuscular	34	18.9
Functional Neurological	25	13.9
Muscle Tension Voice Disorder	44	24.4

Diagnostic distribution across the 180 classifications assigned by four raters (45 unique patients).

## Discussion

### Barriers to the translation of automatic voice disorder detection

This study supports the assertion frequently made by ML researchers that diagnostic inconsistency is a key obstacle to developing reliable voice classification models (2, 8). Our findings confirm that inter-rater agreement was below “good” (*κ* < 0.61) across most diagnostic layers, even when clinicians used a shared classification framework and underwent structured training. This suggests that the lack of standardization in clinical practice is not merely theoretical but persists even under controlled conditions. Furthermore, the absence of a universally adopted classification system likely contributes to the variability of clinical and research datasets. We provide a step toward addressing this gap by introducing and evaluating a multilayer diagnostic framework. Still, the modest reliability results underscore the difficulty of achieving diagnostic consistency, particularly for perceptual and inferential categories.

Although many ML models have achieved strong performance in detecting disordered voices using acoustic or endoscopic features, their real-world utility is severely limited by the variability of the diagnostic inputs they are trained on (1, 6). As our results show, while clinicians demonstrate high intra-rater reliability and strong inter-rater agreement at broader diagnostic levels, agreement declines substantially for perceptually defined or ambiguous conditions. This diagnostic variability, particularly evident in categories like muscle tension voice disorder (MTVD) and vocal fold paresis. It undermines the integrity of the training data used to build ML models and helps explain why such models have not been translated reliably into clinical practice.

This insight reframes the central challenge in voice-AI development. The issue is not that ML models are underpowered, but that the diagnostic ecosystem they rely on is fragmented and inconsistently applied. Model predictions cannot be trusted to perform consistently across settings without stable, reproducible diagnostic inputs from clinicians.

### There is no universally accepted system for classifying voice disorders

The lack of a universally accepted classification system has long been recognised as a bottleneck in clinical practice and computational research (22, 23). Existing frameworks differ in diagnostic logic, terminology, and structure, preventing consistent use across institutions or disciplines. Our multilayer framework was developed to resolve this issue, offering a clinically intuitive yet computationally structured hierarchy. The framework demonstrated high reliability at upper diagnostic levels, supporting its value in standardising the most foundational diagnostic decisions, such as whether a voice is disordered, and whether its origin is organic or functional.

Given their relatively higher diagnostic agreement, these higher-order decisions, such as distinguishing disordered from non-disordered voice or assigning broad aetiological categories, may be particularly well suited for machine learning. As such, they could represent logical starting points for future ML model development and prospective clinical testing, particularly in applications like triage or broad screening support. As ML models mature, deeper framework levels, specific diagnoses, and structural subtypes may become viable prediction targets, especially with improved input quality and larger datasets.

### Reasons for the voice disorder classification challenge

Our findings validate the study hypothesis: reliability would be highest for broad diagnostic categories and lower for perceptually based or inferential diagnoses. Disorders with clear structural correlates, such as nodules, cysts, or paralysis, were classified consistently. Despite training and standardization, MTVD and mild vocal fold paresis yielded poor inter-rater agreement. This reinforces a core theme: specific diagnoses remain inconsistently applied because they lack objective markers and rely on perceptual interpretation. This impairs clinical communication and treatment planning and undermines model generalisability when such conditions are included in training sets.

In the case of MTVD, disagreement likely stems from varied thresholds for identifying hyperfunction, diverse conceptual models across specialties, and exclusion-based diagnostic logic. For vocal fold paresis, subtle asymmetries or borderline findings create room for divergent interpretations. These findings mirror prior literature and suggest that variability is not due to lack of expertise but to the inherent ambiguity of these diagnostic categories (8, 24). Until more objective diagnostic tools (e.g., EMG for paresis, tension metrics for MTVD) are integrated into voice assessment, such labels will continue to be applied inconsistently.

### Developing a robust multilayer diagnostic framework for ML

The hierarchical structure of the proposed framework accommodates diagnostic ambiguity by enabling classification at the most reliable level supported by available data. While this reflects clinical reasoning where clinicians often work from general to specific diagnoses, it also offers important advantages for machine learning. Although ML models can directly classify specific diagnoses (Level 4), their performance is limited by the quality of available training labels (25). Our findings show clinicians agree more consistently at broader diagnostic levels, such as disorder presence or primary aetiology. This suggests an opportunity to improve existing voice datasets: by mapping imprecise or inconsistent labels to broader levels in the hierarchy, researchers can create more reliable training data without reannotating entire datasets. Moreover, hierarchical post-processing of ML outputs can improve interpretability, offering clinically meaningful insights from otherwise complex prediction tasks.

Strong intra-rater reliability and similar inter-rater performance across otolaryngologists and speech-language pathologists suggest that the framework is intuitive, adaptable, and interdisciplinary. These qualities make it well-suited for use in both research and real-world clinics. In ML contexts, the framework allows data to be labeled flexibly and consistently, reducing noise and increasing the reproducibility of algorithm training.

### Diagnosis needs to be based on a multidimensional assessment

The framework captures the multidimensional nature of real-world voice diagnostics by anchoring classification in multimodal inputs, patient case history, perceptual voice assessment, acoustic and aerodynamic analysis, and videostroboscopic imaging. This allows the system to support diagnostic decisions grounded in a complete clinical picture, rather than relying solely on one modality. However, our results also illustrate the limitations of this approach: even with comprehensive clinical data, certain diagnoses remain difficult to apply reproducibly.

This highlights the need for integrating additional tools into diagnostic practice. Technologies such as laryngeal EMG for neuromuscular disorders, structured visual-perceptual scoring systems, or acoustic correlates of hyperfunction could complement perceptual evaluation and enhance reliability (26, 27). In addition, clinician training protocols, including calibration exercises and case-based exemplars, may improve agreement across users and settings. The framework offers a structure for systematically incorporating such enhancements.

### Need for detailed and consensus-driven diagnostic criteria for voice disorders

The findings of this study reinforce the clear and current need for more definitive and detailed diagnostic criteria to be established for different voice disorders. Whilst the Classification Manual for Voice Disorders-I (28), provides a framework for classifying various voice disorders, using American Psychiatric Association's Diagnostic Statistical Manual (DSM) structure, it does not include the emerging diagnostic differentiations of disorders (for example functional neurological disorder) (9) nor, include detailed criteria for difficult to diagnose conditions such as MTD and VFP, and has not been developed with via a wide international consensus process. A foundational reference that is regularly revised, such as the DSM-V (29), is needed in the field to improve diagnostic accuracy and specificity across voice disorders globally.

### Limitations and future directions

Our study was limited by its single-centre design, small sample size, and use of experienced clinicians, which may overestimate reliability compared to broader clinical populations. Diagnostic decisions were made without confirmatory testing such as EMG or aerodynamic profiling, which is particularly relevant in ambiguous cases like paresis or MTVD. The distribution of cases was artificially balanced across categories and may not reflect real-world prevalence patterns. In addition, while we demonstrated feasibility and rater consistency, we did not assess the time burden or usability of the classification system in a routine clinic setting. Moreover, not all diagnostic labels defined within the five-level framework were represented in our sample. Some rare or highly specific voice disorder categories were not tested for inter- or intra-rater agreement, limiting the generalisability of our findings across the entire diagnostic taxonomy.

Future research should focus on validating the framework in multicentre studies, including clinicians with varying levels of voice expertise and broader patient populations. Specific effort should be directed toward improving reliability in categories where agreement was weakest, possibly through more precise operational definitions, integrating quantitative metrics, or developing decision aids and more targeted training. Prospective use of the framework in clinical settings, coupled with analysis of inter-rater concordance over time, would help assess its real-world impact and refine its design further.

Another critical next step is the construction of large, expert-labelled datasets using this framework. Such datasets could be used to train hierarchical ML models, enable multiclass classification, and provide benchmarks for new algorithm development. Public availability of these datasets would accelerate research, foster collaboration, and facilitate meaningful comparisons between modelling approaches. Integrating the framework into electronic health records or digital diagnostic platforms could further streamline its use and enhance standardization across institutions.

Lastly, adopting structured diagnostic frameworks like this is foundational for developing trustworthy, explainable AI in voice medicine. By aligning model training labels with structured clinical reasoning, the framework facilitates interpretability, an essential quality for clinical acceptance of ML tools. In this way, standardized diagnostic input improves clinical reliability and lays the groundwork for safe and effective AI deployment in the future.

## Conclusion

This study presents the first evaluation of a standardized, multilayer voice disorder classification framework designed to improve diagnostic reproducibility and support the development of machine learning applications. The framework demonstrated high inter- and intra-rater reliability for clearly defined categories, including structural, organic, and functional neurological voice disorders. In contrast, agreement was markedly lower for perceptually based diagnoses such as muscle tension voice disorder and vocal fold paresis, highlighting persistent challenges in these domains. Importantly, the system was applied consistently across both laryngologists and speech-language pathologists following structured training, supporting its interdisciplinary utility.

Whilst there are invaluable and essential contributions that ML model testing and development can, and will make to informing what data and specific models are best to use for diagnostic purposes, our findings suggest that variability in diagnostic input, not algorithmic design, is a key barrier to successfully translating ML models into clinical practice. By enabling structured, reproducible labelling across diagnostic layers, this framework provides a foundation for both clinical standardization and supervised learning. Future research should focus on external validation across diverse populations, refinement of ambiguous diagnostic categories, and implementation within large-scale, labelled datasets. Standardising diagnostic processes is essential for improving clinical communication, enabling multi-institutional collaboration, and accelerating the development of trustworthy, AI-powered voice diagnostic tools.

## Data Availability

Deidentified individual participant data that underlie the results reported in this article, along with the study protocol and statistical analysis plan, will be made available beginning 6 months after publication and ending 5 years after publication. Data will be available to researchers who provide a methodologically sound proposal for use in achieving the goals of the approved project. Proposals should be directed to cate.madill@sydney.edu.au. To gain access, data requestors will need to sign a data access agreement.
