# Supplementary material for: Classifying voice disorders for machine learning: a pilot study using the USVAC-C2025 diagnostic framework
Source: Front Digit Health. 2026 Jun 23;8:1752356. doi: 10.3389/fdgth.2026.1752356 (PMC13338672; doi:10.3389/fdgth.2026.1752356)
Supplement: Supplementary file 1 [file supplementaryfile1.docx]

# **Supplementary Table 1. Multilayer Voice Disorder Diagnostic Framework**

Supplementary Table 1. This framework illustrates the five-level hierarchical classification system standardizing voice disorder diagnosis across clinical and machine learning contexts. Each level builds on the previous, supporting broad and specific labelling depending on diagnostic certainty and input completeness.

| Diagnostic Level / Category | Definition | Sources |
| --- | --- | --- |
| L0: Normal vs Abnormal | Binary classification indicating whether the patient's voice is perceptually and/or instrumentally assessed as within normal limits or disordered. | Roy et al., 2013 (1); Eadie et al., 2005 (2) |
| L1: Etiological Category | Classification is based on the primary underlying cause: organic (structural or neuromuscular), functional neurological, or muscle tension disorder. | Morrison & Rammage, 1986 (3) ; Morrison & Rammage (1993) (4); Verdolini et al 2005 (5) ; Payten et al., 2022  (6) |
| L2: Organic Subtype | Subdivision of organic voice disorders into structural (e.g., lesions, oedema, scarring) and neuromuscular (e.g., vocal fold paralysis, paresis) disorders. | Rosen & Murry, 2000 (7)  ; Isseroff et al., 2016 (8) |
| L3: Diagnostic Grouping | Aggregated diagnostic classes within each subtype include benign lesions, inflammatory laryngitis, neurogenic paresis, and functional aphonia. | Verdolini et al., 2005 (5); Morrison & Rammage, 1986 (3); Altman et al., 2007 (9) |
| L4: Specific Diagnosis | Most granular diagnostic labels are assigned based on a comprehensive assessment (e.g., Reinke's oedema, vocal fold nodules, spasmodic dysphonia). | Morrison et al., 1993 (3) ; Verdolini et al (5)  ; Payten et al., 2022  (6) |

1.  Roy N, Barkmeier-Kraemer J, Eadie T, Sivasankar MP, Mehta D, Paul D, Hillman R. Evidence-based clinical voice assessment: a systematic review. American Journal of Speech-Language Pathology. 2013;22(2):212-226. <https://doi.org/10.1044/1058-0360(2012/12-0014)>

2. Eadie TL, Doyle PC. Classification of dysphonic voice: acoustic and auditory-perceptual measures. J Voice Off J Voice Found. 2005 Mar;19(1):1–14. doi:10.1016/j.jvoice.2004.02.002 PubMed PMID: 15766846.

3. Morrison MD, Nichol H, Rammage LA. Diagnostic criteria in functional dysphonia. The Laryngoscope. 1986 Jan;96(1):1–8. doi:10.1288/00005537-198601000-00001 PubMed PMID: 3941573.

4. Morrison MD, Rammage LA. Muscle misuse voice disorders: description and classification. Acta Otolaryngol (Stockh). 1993 May;113(3):428–34. doi:10.3109/00016489309135839 PubMed PMID: 8517149.

5. Verdolini, K., Rosen, C. A., & Branski, R. C. (Eds.). (2012). Classification Manual for Voice Disorders-I. New York, NY: Psychology Press. <https://doi.org/10.4324/9781410617293>".

6. Payten CL, Chiapello G, Weir KA, Madill CJ. Frameworks, Terminology and Definitions Used for the Classification of Voice Disorders: A Scoping Review. J Voice. 2022. Located at: Scopus. doi:10.1016/j.jvoice.2022.02.009

7. Rosen CA, Murry T. Nomenclature of Voice Disorders and Vocal Pathology. Otolaryngol Clin North Am. 2000 Oct 1;33(5):1035–45. doi:10.1016/S0030-6665(05)70262-0

8. Isseroff TF, Parasher AK, Richards A, Sivak M, Woo P. Interrater Reliability in Analysis of Laryngoscopic Features for Unilateral Vocal Fold Paresis. J Voice Off J Voice Found. 2016 Nov;30(6):736–40. doi:10.1016/j.jvoice.2015.08.018 PubMed PMID: 26471809.

9. Altman KW. Vocal fold masses. Otolaryngol Clin North Am. 2007 Oct;40(5):1091–108, viii. doi:10.1016/j.otc.2007.05.011 PubMed PMID: 17765697.
